# A Thin Layer of Decellularized Porcine Myocardium for Cell Delivery

**DOI:** 10.1038/s41598-018-33946-2

**Published:** 2018-11-01

**Authors:** Mickey Shah, Pawan KC, Katherine M. Copeland, Jun Liao, Ge Zhang

**Affiliations:** 1 Integrated Bioscience Program, The University of Akron, Akron, OH 44325-0302 USA; 2 Department of Biomedical Engineering, The University of Akron, Akron, OH 44325-0302 USA; 3 Department of Bioengineering, University of Texas at Arlington, Arlington, Texas 76019 USA

## Abstract

Decellularized porcine myocardium has shown many benefits as a cell delivery scaffold for cardiac therapy. However, using full thickness decellularized myocardium as cardiac patch may lead to poor viability and inhomogeneous distribution of delivered cells, due to perfusion limitations. In this study, we explored the feasibility of decellularized porcine myocardial slice (dPMS) to construct a vascularized cardiac patch for cell delivery. Decellularized porcine myocardium was sliced into thin layers (thickness~300 µm). Adipose-derived Stem cells (ASCs) obtained from rat and pig were seeded on dPMS. The viability, infiltration, and differentiation of seeded ASCs were examined. The mechanical properties of dPMSs of various thickness and native myocardium were tested. We noticed dPMS supported attachment and growth of rat and pig ASCs. Both rat and pig ASCs showed high viability, similar patterns of proliferation and infiltration within dPMS. Rat ASCs showed expression of early-endothelial markers followed by mature-endothelial marker without any additional inducers on dPMS. Using rat myocardial infarction model, we delivered ASCs using dPMS patched to the infarcted myocardium. After 1 week, a higher number of transplanted cells were present in the infarcted area when cells were delivered using dPMS versus direct injection. Compared with MI group, increased vascular formation was also observed.

## Introduction

Cardiac patches have shown many advantages in delivering the required large amount of stem cells to repair or replace the lost cardiomyocytes after acute myocardial infarction (MI). It has been reported that approximately 1 billion cardiomyocytes are lost in humans during an MI^1,2^. As cardiomyocytes have an extremely limited regenerative capacity, exogenous cell transplants have been conducted to compensate for the lost cardiomyocytes and improve the compromised heart function^3^. In various clinical trials, cells varying from 1–200 million have been delivered to the heart to fulfill functional recovery^4–6^. Unfortunately, the retention rate of the delivered cells has been found to be extremely low via traditional injection^7^. To increase the cell delivery capacity as well as area coverage, injecting cells at 5–6 points within and around the infarcted area has been utilized by many groups. However, mounting evidence has revealed that multi-injections of large amount of cells into the infarcted heart causes the heterogeneous distribution of the cells, which may increase the possibility of ventricular arrhythmias^8–10^. As an alternative approach for cell delivery, cardiac patches can deliver a significant amount of cells, covering the entire injured region of the heart in a homogeneous manner^11–14^.

An ideal cardiac patch should closely mimic the natural microenvironment hosting the various types of cardiovascular cells. The importance of microenvironment on cell survival, growth, and function has been proven by numerous studies over the past decade^15–17^. Both physical (e.g. stiffness, microstructure) and chemical (e.g. composition, growth factors) characteristics of the microenvironment play significant roles on the cells. Decellularized cardiac tissue has great potential to make an ideal cardiac patch. Cardiac extracellular matrix (ECM) has a unique 3D microstructure and complicated chemical composition containing multiple collagen isoforms and various proteins such as elastin, laminin, fibronectin, hyaluronan, glycosaminoglycans (GAGs), chondroitin sulfate proteoglycans, heparin sulfate, and different growth factors^18^. By optimizing the decellularization methods, researchers can preserve the perfusable vascular tree, ultrastructure of the ECM and retain growth factors after porcine heart decellularization^19,20^. Additionally, decellularized cardiac ECM has been shown to facilitate the cardiac differentiation of stem cells. When human multi-potential cardiovascular progenitor cells were used to repopulate the whole decellularized mouse heart, the seeded cells were found to differentiate into various cardiovascular cell types *in situ* with high efficiency^21^. Our previous studies have also shown the facilitated vascular differentiation of hMSCs by hydrogels made of decellularized porcine cardiac ECM^22^. The high biomimicry nature of cardiac ECM makes it an optimal scaffold for cardiac tissue engineering application.

Recently decellularized porcine ECM has gained increasing interest in cardiovascular research due to similarities between porcine and human heart ECM in terms of their composition, microstructure, vascular tree distribution, and mechanical properties^23–25^. However, direct using full thickness of decellularized porcine ECM as cardiac patch for cell delivery will have major foreseen problems. First, homogenous cell distribution will be hard to achieve in full thickness porcine decellularized ECM. It has been widely reported that cells seeded in the center of thick scaffold have very low viability due to their insufficient access to oxygen and nutrients^26,27^. Second, the weight of full thickness decellularized porcine ECM may increase cardiac afterload when applied as a cardiac patch to the injured myocardium, which could negatively contribute to the LV remodeling after MI. Last but not least, patching full thickness decellularized porcine ECM to heart may alter the local geometry and mechanical properties and therefore affect normal cardiac function.

In this study, we explored the feasibility of using decellularized porcine myocardial slice (dPMS) to construct a vascularized cardiac patch for cell delivery. We hypothesize that a thin layer of decellularized porcine myocardium will promote cell attachment, growth, homogeneous distribution and vascular differentiation of stem cells. Decellularized porcine myocardium was sliced into a thin layer (thickness ~300 µm) for cell seeding. Adipose derived Stem Cells (ASCs) were chosen in this study to recellularize dPMS for the following reasons. First, ASCs are obtained from abundant adipose tissue which make them a practical autologous stem cell source for cell therapy^28,29^. Second, ASCs have been shown to promote angiogenesis and are widely used in treating ischemic tissues^30–32^. Last, ASCs have demonstrated many benefits when used to treat cardiovascular diseases, such as their capability of differentiating into cardiomyocytes, vascular smooth muscle cells, endothelial cells, secretion of potent paracrine factors to promote neovascularization, reducing apoptosis, and inhibiting fibrosis^33^. Both porcine and rat ASCs were used to recellularize dPMSs to elucidate any differences in cell-ECM interactions caused by various species. After seeded to dPMS, the viability, distribution and vascular differentiation of ASCs within the dPMS were assessed. The retention of the delivered cells and vascularization potential of dPMS were also assessed *in vivo* using rat acute myocardial infarction model. Additionally, the effects of dPMS thickness on scaffold mechanical properties were examined.

## Results

### Isolation and Characterization of Rat and Pig ASCs

**Figure 1:**
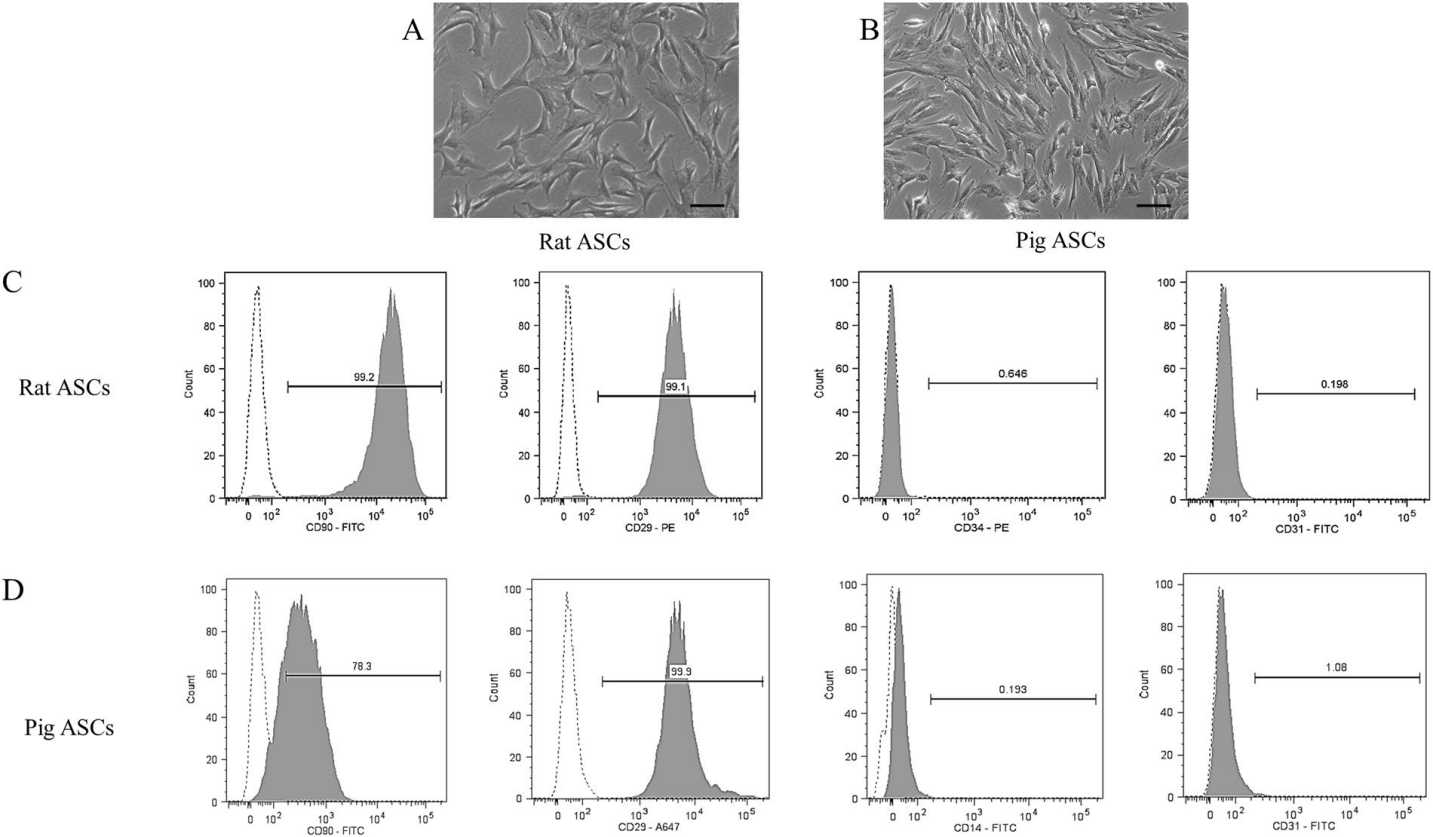
Characterization of the isolated rat and pig ASCs: Morphology of the isolated rat ASCs (**A**) and pig ASCs (**B**) at passage 2. Flow cytometer analysis of the cell marker expressions for the isolated rat ASCs (**C**) and Pig ASCs (**D**). Scale bar 100 µm.

SVF from white adipose tissue was cultured normally to obtain the ASCs from rat and pig sources. Colony formation in the culture flask was observed after 3–5 days. The rat and pig ASCs were obtained upon culturing and expansion of the colonies. Regardless of species, the obtained ASCs showed typical fibroblast-like morphology (Fig. 1A,B) as reported by many other groups^34,35^. Flow cytometry analysis was performed to further confirm the phenotype of the obtained ASCs. As demonstrated by many groups, we selected two positive mesenchymal stem cell markers (CD90 and CD29) and lack of hematopoietic lineage marker (CD34 and CD14) and endothelial marker (CD31) for analysis^36,37^. Due to unavailability of anti-CD34 marker for pig ASCs, we selected anti-CD14 marker. Our data show that the obtained rat ASCs highly expressed CD90 (99.2%) and CD29 (99.1%). While the expressions for CD34 (0.646%) and CD 31(0.198%) were extremely low (Fig. 1C). Similarly, CD90 (78.3%) and CD29 (99.9%) were highly expressed in the isolated pig ASCs and the expression of CD31 (1.08%) and CD14 (0.193%) were extremely low (Fig. 1D).

### Characterization of Decellularized Porcine Myocardium Slices

**Figure 2:**
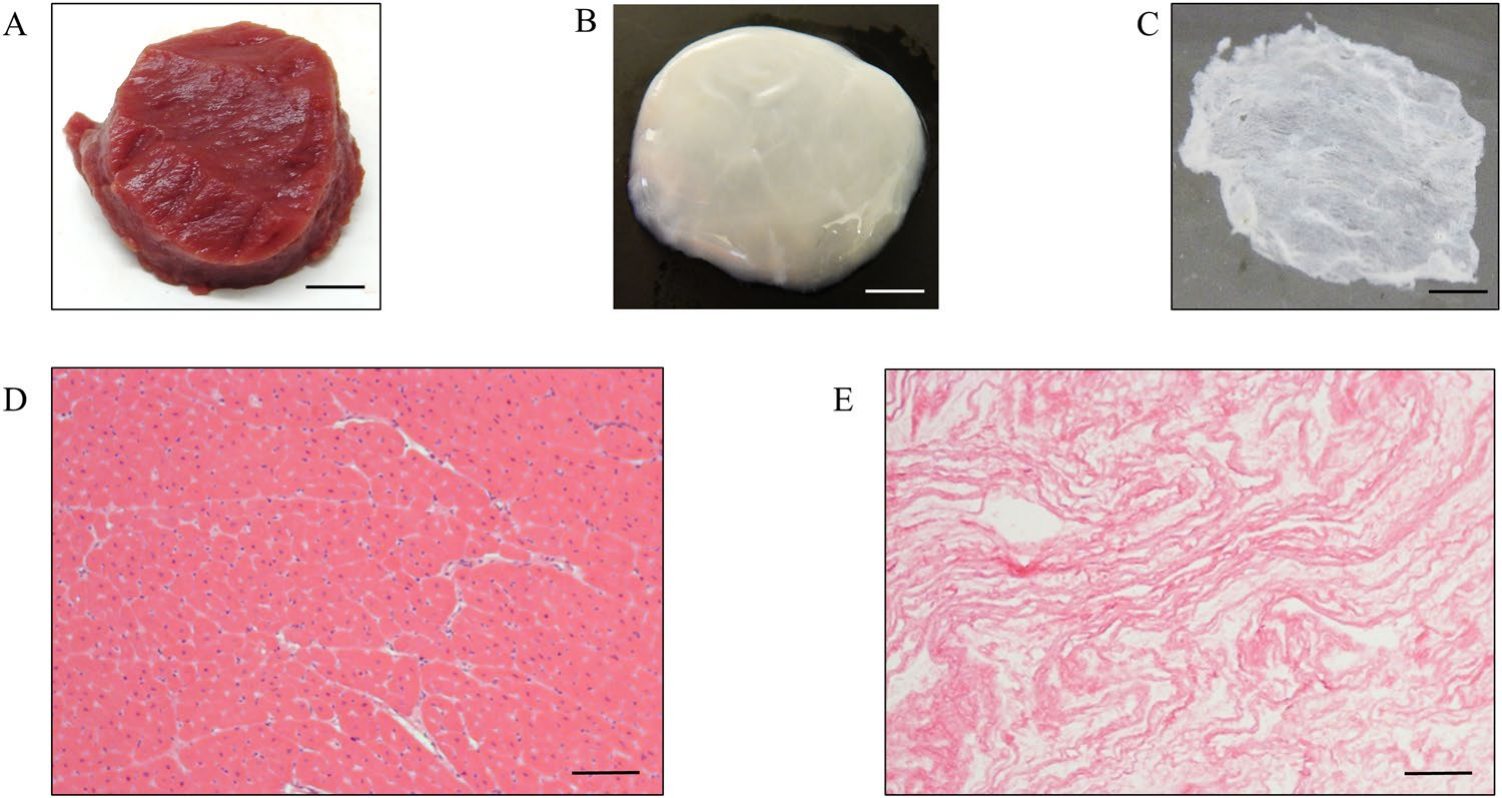
Fabrication of the decellularized porcine myocardium Slice: Native myocardial tissue before (**A**) and after (**B**) the decellularization process. The decellularized porcine myocardium was cut into 300 µm decellularized porcine myocardium slices (**C**). H & E staining of native myocardial tissue (**D**) and decellularized myocardial tissue (**E**). Scale bars: 4 mm in A, B, and C; 100 µm in D and E.

Native myocardial tissues were observed to turn white during the decellularization process, indicating the removal of cellular component from the tissues (Fig. 2A,B). Our established decellularization protocol has proven to significantly reduce the DNA content of the native tissue and still maintain the presence of ECM proteins such as collagen and GAGs (see Supplementary Fig. S3)^22^. dPMS (300 µm thickness) were successfully obtained using cryosection with retained physical form (Fig. 2C). H&E staining of the dPMS confirmed sufficient decellularization, showing no cellular presence and well preserved ECM as compared to native myocardium tissue (Fig. 2D,E).

### Cell Viability and Proliferation on dPMS

**Figure 3:**
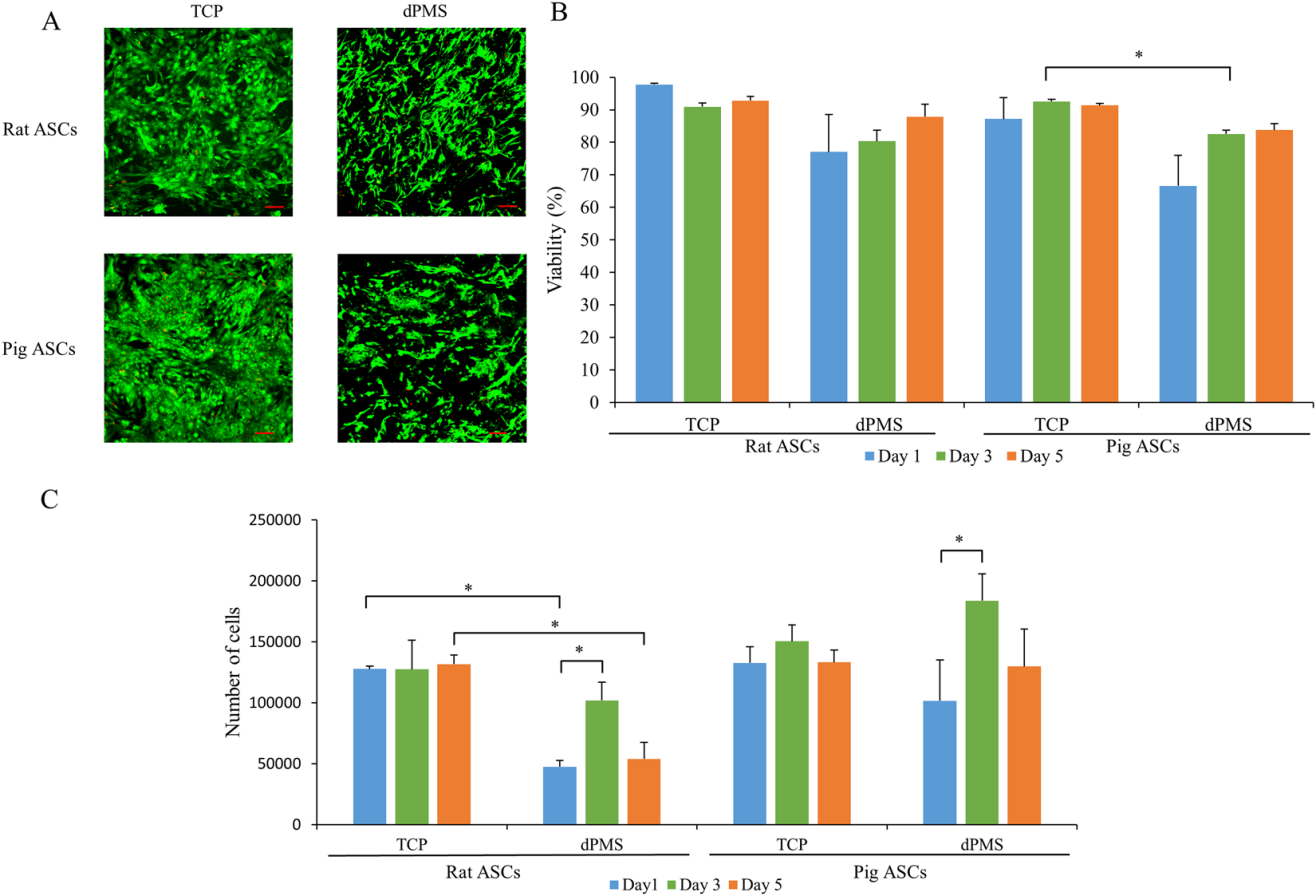
Cell viability and proliferation of Rat and Pig ASCs on dPMS. (**A**) Live and dead cells on dPMS and TCP after 1 day. Scale bar 100 µm. (**B**) Percent viable live cells after seeding on the dPMS on day 1, 3, and 5. (**C**) Cell proliferation of rat ASCs and pig ASCs seeded on dPMS at day 1, day 3 and day 5. Data were expressed as means ± SD (n = 3 for each sample). *Represents the statistical significant difference with p value < 0.05.

Rat and pig ASCs (200,000/slice) were seeded onto dPMSs and their viability and proliferation were tested and compared with the cells seeded on a tissue culture plate (TCP) under the same culture conditions. 24 hours after seeding, both rat and pig ASCs were observed to be attached to the dPMSs and spread in an elongated morphology (Fig. 3A). The cells seeded on dPMSs had less confluency as compared with cells seeded on TCP indicating the infiltration of cells into the dPMSs. The cell viability of rat ASCs on TCP was 98% ± 0.4%, 91% ± 1.1% and 93% ± 1.3% for days 1, 3 and 5. The viability of pig ASCs on TCP was 87% ± 6.6%, 93% ± 0.7%, and 91% ± 0.6% for days 1, 3 and 5. Rat ASCs viability on 300 µm dPMS was 77% ± 11.4% on day 1, 80% ± 3.4% on day 3, and 88% ± 3.9% on day 5. Pig ASCs viability on 300 µm dPMS was 67% ± 9.4% on day 1, 83% ± 1.1% on day 3, and 84% ± 1.9% on day 5. There was no significant difference when comparing the viability of rat ASCs cultured on dPMSs with those cultured on TCP on days 1, 3, and 5. The viability of pig ASCs cultured on dPMSs were significantly lower than those culture TCP on day 3, but no significant difference was found on days 1 and 5.

The proliferation of rat and pig ASCs was examined on days 1, 3, and 5 after seeding onto dPMSs. After initial cell seeding, rat and pig ASCs attached to the dPMSs at different efficiencies. Approximately 26.5% ± 2.5% of rat ASCs and 50.8% ± 16.8% of pig ASCs were found within the dPMS on day 1. Number of rat ASCs attached on dPMS on day 1 (26.5% ± 2.5%) were significantly lower than cultured on TCP at day1 (63.8% ± 1.2%) (See Supplementary Fig. S1). The majority of unattached ASCs sunk to the bottom of the TCP and kept growing there. From day 1 to day 3, both rat and Pig ASCs proliferated significantly within the dPMSs (Fig. 3C). The number of rat and pig ASCs within the dPMS increased ~2.1 fold and ~1.8 fold respectively when comparing the number from day 3 to day 1. The total cell numbers within dPMSs did not change significantly from day 3 to day 5 for both rat and pig ASCs.

### Cell Infiltration on dPMS

**Figure 4:**
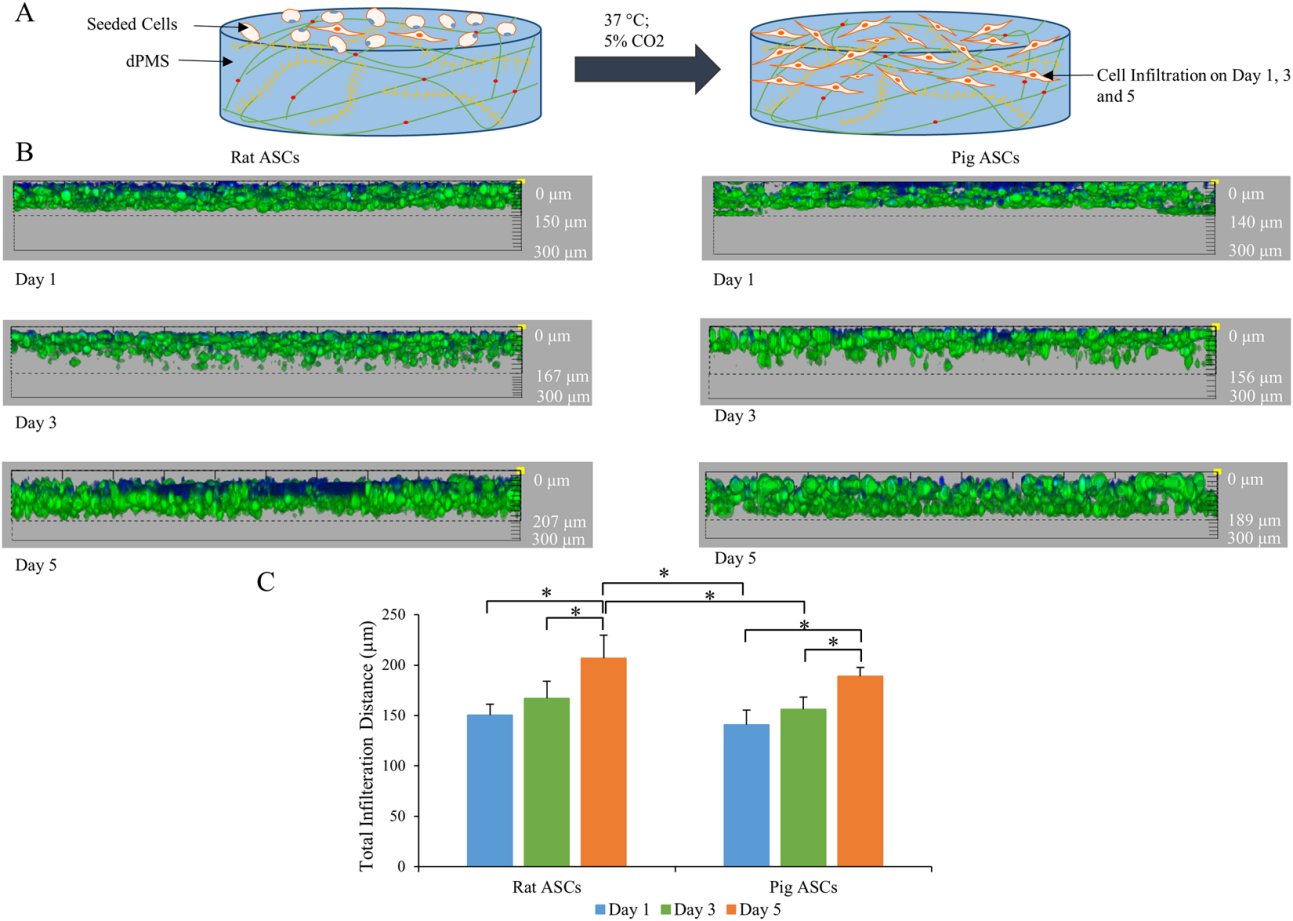
Infiltration of Rat and Pig ASCs on decellularized porcine myocardium slices. (**A**) Schematic diagram of the experiment procedure showing lateral cell infiltration in dPMS. (**B**) Z- stack side view of the infiltrated rat and pig ASCs in 300 µm dPMS. (**C**) Migration distance of rat and pig ASCs seeded on dPMSs after 1, 3, and 5 days culture. *Represents statistically significant difference between experiment groups with p value < 0.05.

The infiltration of rat and pig ASCs within the dPMSs was assessed after days 1, 3, and 5 of cell seeding (Fig. 4A). Confocal Z- stack images confirmed the presence of cells at various planes for both rat and pig ASCs and showed the overall distribution of cells within the dPMSs (Fig. 4B). Rat and pig ASCs demonstrated a very similar infiltration pattern (Fig. 4C). Cells infiltrated into the dPMS after seeding and kept migrating to the other side of the dPMSs. The infiltration distance was found to be significantly longer on days 3 and 5 as compared to day 1. On day 5, the ultimate infiltration distance for rat ASCs was 207 ± 22.5 µm and for pig ASCs was 189 ± 6.2 µm.

### Cardiovascular Differentiation of Rat and Pig ASCs on dPMS

**Figure 5:**
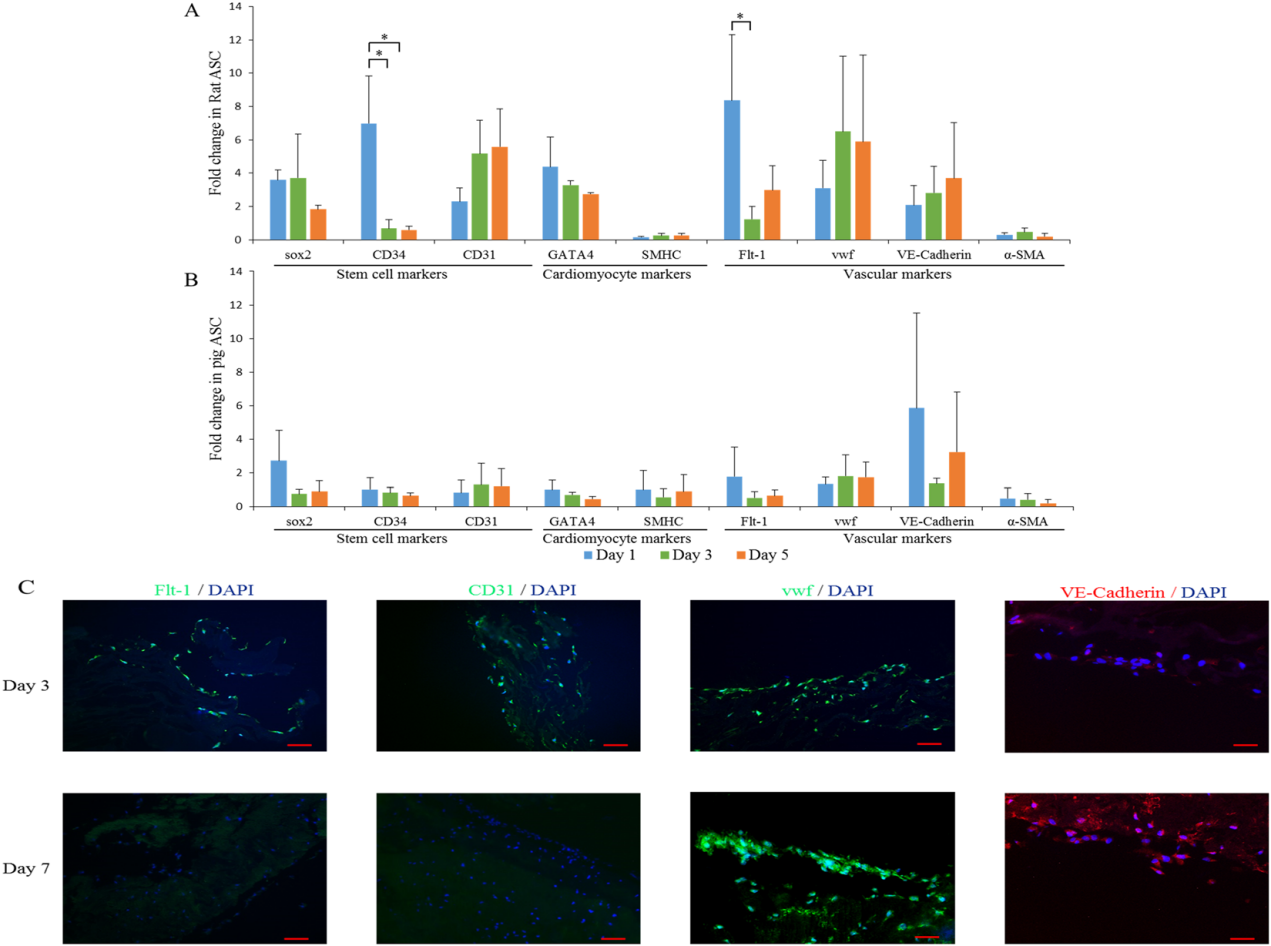
Gene expression profile of cardiovascular markers in (**A**) rat ASCs and (**B**) pig ASCs after day 1, day 3, and day 5 on 300 µm dPMS. Expression of late endothelial marker (vwf and VE-Cadherin) and early endothelial marker (Flt-1 and CD31) at day 3 and day 7 for rat ASCs. Scale bar 50 µm. Data were expressed as means ± SD (n = 3 for each sample). *Represents the statistically significant difference with p value < 0.05.

After being cultured for 1, 3, and 5 days on dPMSs, the relative gene expression profile of rat and pig ASCs was analyzed using qRT-PCR for stem cell markers (sox2, CD31, and CD34), cardiomyocyte markers (GATA-4 and sMHC) and vascular cell markers (α-SMA, Flt-1, vwf, and VE-cadherin). For pig ASCs, none of the examined genes showed significant expression changes (Fig. 5B). For rat ASCs, the expressions of CD34 and Flt-1 were significantly upregulated on day 1 as compared to *in vitro* culture and then significantly downregulated on day 3. Other genes did not show significant changes. However, a trend of increased expression of CD31, vwf, and VE-Cadherin was observed (Fig. 5A). Immunofluorescence staining confirmed this transient expressions of early endothelial cell markers (CD31 and Flt-1) for rat ASCs on day 3 and the increased expression of mature endothelial cell markers (vwf and VE-cadherin) on days 3 and 7 (Fig. 5C).

### Biaxial Mechanical Behavior of dPMS

**Figure 6:**
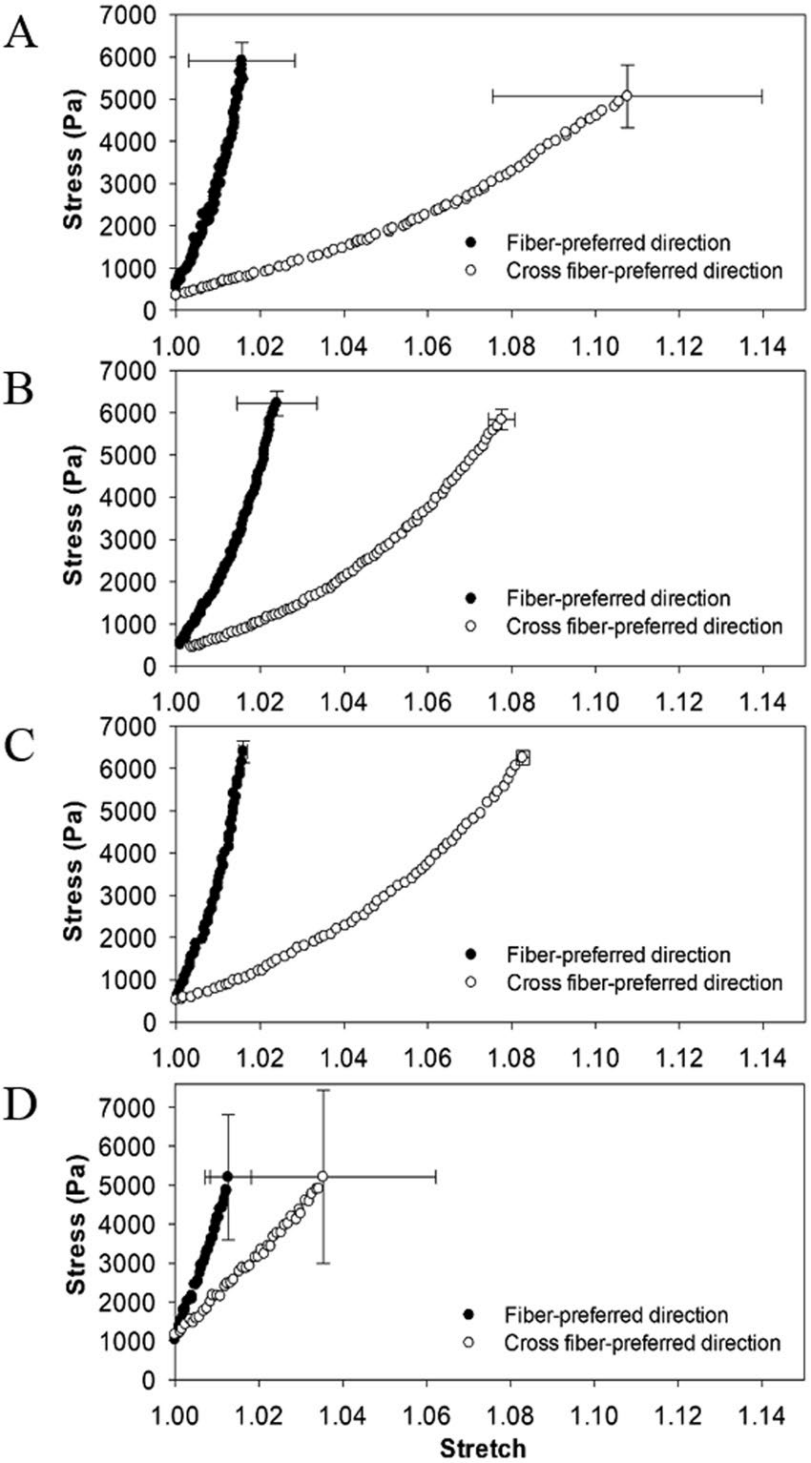
The biaxial mechanical behavior of native cardiac slices of 900 µm thickness (**A**), acellular cardiac ECM slices of 900 µm thickness (**B**), acellular cardiac ECM slices of 600 µm thickness (**C**) and acellular cardiac ECM slices of 300 µm thickness (**D**).

The biaxial mechanical behavior of native cardiac slices with a thickness of 900 µm and dPMS with thickness of 900 µm, 600 µm, and 300 µm are shown in Fig. 6. The biaxial curves are plotted as stress versus stretch along both the fiber-preferred direction and the cross fiber- preferred direction. Our data demonstrated that the dPMS with the thickness of 900 µm and 600 µm (Fig. 6B,C) maintained an overall nonlinear and anisotropic mechanical behavior similar to the native cardiac slices of 900 µm thickness (Fig. 6A). The 900 µm and 600 µm dPMSs were stiffer in stress-stretch behavior when compared with the native 900 µm cardiac slices (Table 1), reflecting the collagen network dominant properties of the acellular ECM. This overall trend was consistent with what was observed in thick acellular cardiac ECM^38–40^. We noticed that the 300 µm dPMS experienced a loss of nonlinearity and extensibility, as well as a decrease of anisotropy (Fig. 6D).

**Table 1:** Extensibilities and maximum tensile moduli along fiber-preferred direction and cross fiber-preferred direction at 5 N/m equibiaxial tension.

Sample	Extensibilities		Maximum Tensile Moduli (Pa)	
	Fiber-preferred	Cross Fiber-preferred	Fiber-preferred	Cross Fiber-preferred
Native slices of 900 µm	1.0153 ± 0.0122	1.1087 ± 0.0308	440,483 ± 304,225	62,876 ± 8,055
Acellular slices of 900 µm	1.0240 ± 0.0095	1.0760 ± 0.0010	406,741 ± 109,124	118,291 ± 5,513
Acellular slices of 600 µm	1.0180 ± 0.0010	1.0827 ± 0.0015	534,010 ± 19,480	106,777 ± 3,194
Acellular slices of 300 µm	*1*.*0145* ± *0*.*0027*	*1*.*0352* ± *0*.*0270*	*363*,*744* ± *189*,*049*	*180*,*036* ± *96*,*466*

Data were expressed as means ± SD.

### *In vivo* Cell Delivery using dPMS

**Figure 7:**
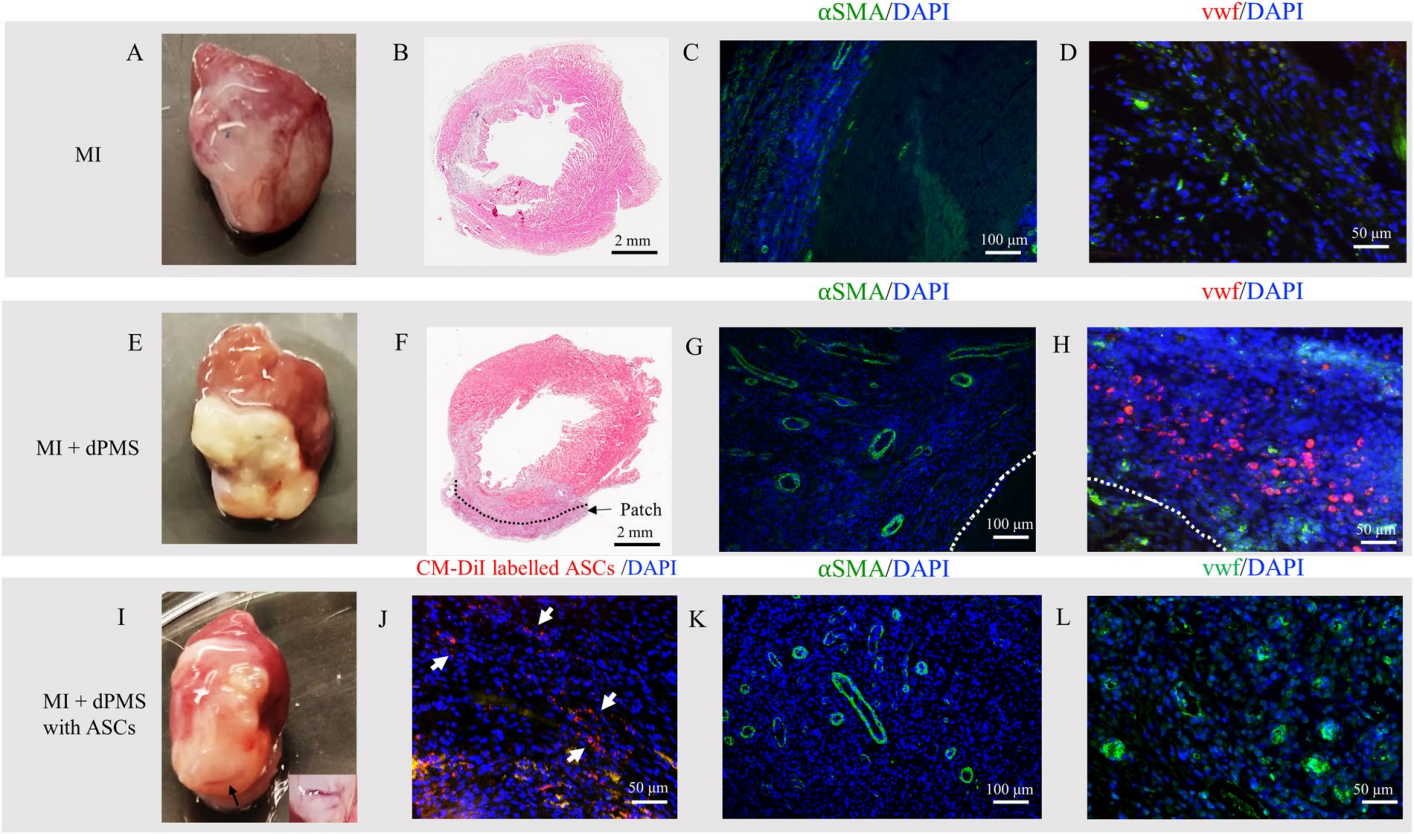
ASC delivery using dPMS and *in vivo* evaluation. Acute MI was induced by LAD ligation in rat (**A**). After 1 week of implantation, dPMS remained on the surface of the infarcted myocardium (**E**,**I**). The harvested heart sections were evaluated by hematoxylin/eosin (H&E) staining (**B**,**F**). Delivered ASCs were labelled with CD-DiI and identified in implanted dPMS (**J**). Vessel formation was observed within the dPMS used for ASC delivery (**I**) The insert is a close up of the vessel branches in the dPMS. The expressing of vascular markers αSMA (**C**,**G**,**K**) and vwf (**D**,**H**,**L**) were detected using immunostaining. Cell nuclei were counterstained with DAPI (blue).

Rat acute MI was successfully induced by ligation of the Left Anterior Descending artery (LAD) that was confirmed by color change of left ventricle wall from red to white (Fig. 7A). Loss of cardiac cells and thinning of the left ventricle wall were observed 1 week post-MI (Fig. 7B). Transplanted dPMS (with and without delivered ASCs) formed tight adhesion to the myocardium 1 week after transplantation, and LV wall thickness of rat myocardium was increased by the attached dPMS (Fig. 7E,F,I). Host cells (DAPI+ cells) infiltrated the implanted acellular dPMS after 1 week and were associated with vascular structures within the dPMS (Fig. 7G,J,K). Labelled ASCs were found present after 1 week when transplanted using dPMS (Fig. 7J). We found significantly more cells present in the infarcted myocardium when delivered using dPMS versus direct injection (see Supplementary Fig. S6). Compared with the infarcted myocardium from the control group (MI without dPMS transplantation), ingrowth of host vasculature vessel was clearly observed within the dPMS and was confirmed by the high expression of vascular markers (α-SMA and vwf). (Fig. 7C,D,G,H,K,L). We found a higher number of total vessels per mm^2^ area in the infarcted myocardium in the group of MI treated by dPMS delivered ASCs than in the control group (MI only) at week 1 (see Supplementary Fig. S5).

## Discussion

In this study, instead of using full thickness decellularized porcine myocardium (~2 mm thickness), we chose to exploit a thin layer of dPMS (300 µm thickness) as a cell-delivering platform. It is well known that scaffold thickness affects cell viability^41,42^. Within the body, most cells are found no more than 100–200 µm from the nearest capillaries for sufficient diffusion of oxygen and nutrients^27^. When cells are seeded in an avascular scaffold, they rely on direct oxygen diffusion to survive. Therefore, a scaffold thicker than a few hundred microns could cause significant cell death, especially in the central core of the scaffold^43^. The limited oxygen diffusion in the thick scaffold could also cause uneven cell distribution. In cardiac applications, heterogeneous cell distribution has been found to trigger arrhythmia^44^. In addition, thick scaffolds may also put extra weight on the pumping heart which could increase the afterload of the injured LV and accelerate the negative remodeling process^45^. From the results of our study, we demonstrated that 300 µm dPMS when recellularized using stem cells, could achieve high cell viability, homogenous cell distribution, support cell growth, and promote cell infiltration within the scaffold. This indicates the great potential of the thin dPMS as a cardiac patch for cell delivery.

Both rat ASCs and pig ASCs have been used to recellularize dPMSs to examine whether species of the cells will affect cell-dPMS interactions. The obtained information is very critical for the planning of next step *in vivo* studies where dPMSs will be used to deliver autologous stem cells to the injured heart using a rat myocardial infarction model. In our study, dPMSs were shown to support the viability, proliferation, and infiltration of both rat and pig stem cells. Pig ASCs, being cells from same species, have shown higher attachment rate to dPMS as compared to rat ASCs (50.8% of pig ASCs attached to dPMS 1 day after cell seeding vs 26.5% of rat ASCs). However, compared with rat ASCs, cell viability of pig ASCs cells is lower on day 1 (66.5% of pig ASCs vs 77% of rat ASCs) and the cell proliferation rate on dPMS from day 1 to day 3 is lower (~1.8 fold for pig ASCs vs ~ 2.1 fold for rat ASCs). Rat ASCs also infiltrated deeper into the dPMS as compared with pig ASCs after 5 days of culture (189 µm of pig ASCs vs 207 µm of rat ASCs). Based on our observations the size of pig ASCs and rat ASCs have no significant difference. We suspect the differences in cell viability, proliferation, and infiltration are caused by cell species.

We also investigated whether dPMS retain the benefits of decellularized porcine myocardium in term of mimicking the chemical and mechanical microenvironment of the cardiac ECM. Our data from this study showed that endothelial and stem cell markers (flt 1 and CD34) were transient increased 1 day after rat ASCs seeded onto dPMS without any adding chemical inducers, suggesting the accelerated endothelial differentiation of rat ASCs by dPMS. Previous studies from our lab also demonstrated the induced endothelial differentiation of human mesenchymal stem cells by dPMSs^46^. These results indicate that dPMS possess the cues of cardiac ECM that would control the vascular differentiation of stem cells. The dPMS directed endothelial differentiation of the stem cell could be exploited to developed vascularized scaffold to improve cell delivery efficiency. We did not find either cardiac differentiation of the rat ASCs (see Supplement Fig. S2) or cardiac and vascular differentiation of the pig ASCs when they were cultured on the dPMSs. We believe this is due to the insufficient stimuli derived from dPMS alone and potency of the cells. Although rat ASCs have been reported to be able to differentiate into cardiomyocytes *in vitro*, either chemical inducer or co-culturing with contracting cardiomyocytes were required for these studies^47^. Compared to rat ASCs, the pig ASCs have been less investigated. Limited reports have been published for cardiovascular differentiation of pig ASCs^48^. The mechanical testing data from this study shows that, compared with dPMSs of 900 µm and 600 µm, the 300 µm thick dPMS experienced a loss of nonlinearity and weakening of mechanical properties. This is likely due to the challenge of maintaining network structure at such a thin thickness.

The promise of using dPMS for cardiac repair was demonstrated from our preliminary *in vivo* studies using rat acute MI model. When patching to infarcted rat myocardium, dPMS demonstrated great engraftment with the host tissue. dPMS was able to tightly attach to rat myocardium and maintain physical integrity 1 week after transplantation. dPMS encouraged the infiltration of host cells which was demonstrated by the wide distribution of DAPI+ cells in the transplanted dPMS. To examine whether these infiltrated cells contained host inflammatory cells, we checked the CD68+ and CD 163+ expressions of these DAPI+ cells. (See Supplementary Fig. S4). The results indicated that most of these cells are macrophages (CD68+) with a considerable amount of M2 macrophages (CD 163+) which has been reported to promote tissue repair^49^. Most importantly, our preliminary *in vivo* data proved that dPMS can be used to deliver cells with high cell retention rate. Increased vascularization could be one of the main mechanisms behind the improved cell retention since vascular structures were found throughout the entire grafted dPMS. The revascularization of decellularized cardiac matrix in myocardial infarcted animals have been observed and reported^50^. For example, neoformation of vessels were found after transplanting a human decellularized pericardium to a swine infarcted heart^51^. These promising results show the potential of using thin dPMS for cell delivery and vascularization in cardiac therapy. In the future, we will examine the *in vivo* functional efficiency and effectiveness using dPMS for MI treatment.

## Methods

See supplementary methods for Hematoxylin & Eosin staining, Flow Cytometry Analysis, and Quantiative Real Time Polymerase Chain Reaction (qRT-PCR) analysis protocol.

### Isolation and Culture of Rat and Pig ASCs

Rat adipose tissue was harvested from euthanized animal in accordance with relevant guidelines, regulations, and approval of the Animal Care and Use Committee (IACUC) at the University of Akron (14-11-14-ZRD). The fresh pig adipose tissue was obtained from a local butcher (Pressler’s Meat, Akron, OH). Rat and pig ASCs were isolated from harvested subcutaneous white adipocyte tissues following the protocol as previously described^52,53^. Briefly, 1 g of abdominal subcutaneous white adipocyte tissue was harvested, minced, and digested with 4 mg/ml type II collagenase (1039 CDU/mg, Sigma-Aldrich, USA) for 1 hour at 37 °C with gentle agitation. The digested cell suspension was then filtered through 100 µm nylon mesh and centrifuged at a speed of 250 g for 5 minutes to obtain stromal vascular fraction (SVF) from the suspension. The SVF was washed thoroughly three times using PBS, resuspended in 5 ml Mesen-Pro RS culture media (Life technologies, USA) and cultured in a cell culture incubator (37 °C; 5% CO_2_) for ~5 days to become 80% confluent. The culture medium was changed every 3 days. Passage 2–5 of ASCs were used in this study.

### Preparation of Decellularized Porcine Myocardium Slices (dPMS)

Porcine myocardium tissues were decellularized following the established protocol^46^. Fresh pig hearts were harvested from a local butcher (Pressler’s Meat, Akron, OH). Square myocardium biopsies (2 cm length × 2 cm width × 1 cm height) were dissected from middle of the anterior left ventricular wall. After a brief wash in DI water to rinse excess blood, myocardium was decellularized by 1% (w/v) sodium dodecyl sulfate (SDS) with 0.5% Penicillin/streptomycin at room temperature for 2.5 weeks with gentle agitation. Fresh SDS was changed every day. At completion of decellularization process, decellularized tissue was washed with 0.01% Triton X-100 for 1 hour and then rinsed with 1X phosphate buffered saline (PBS) for three days and stored finally at −20 °C until use. Decellularized porcine myocardium slices of 300 µm, 600 µm, and 900 µm thickness were obtained by cryosectioning the decellularized tissue embedded in HistoPrep tissue embedding media (Fisher, USA).

### Recellularization of the dPMS

Sterilization of the dPMS was performed with absolute ethanol for 45 minutes followed by at least three washes with sterilized DI water for 15 minutes each. The dPMS were then kept overnight in Mesen-Pro RS culture media. The next day, 200,000 rat or pig ASCs in 30 µl of culture media were seeded on top of the dPMS using a custom stainless steel ring to hold the slices down (inner diameter 10 mm). The system was incubated in a cell culture incubator (37 °C; 5% CO_2_) for 4 hours to facilitate maximum cell attachment. After 4 hours, 2 ml of culture medium was added to slices for cell culture. Cell culture medium was changed every 2 days after recellularization. Cells were labelled with cell tracker CM-DiI dye (Invitrogen) before seeding on top of the dPMS to create the dPMS with labelled ASCs. One day after the seeding, they were used for implantation.

### Cell Viability and Cell Infiltration Analysis

The viability of rat and pig ASCs cultured on dPMS was assessed on days 1, 3, and 5 after cell seeding. The samples were stained with hoechst, calcein AM, and ethidium homodimer-1 (2 mM; Molecular Probes). After washing with PBS, the samples were imaged with a confocal microscope. Three representative areas from each slice were captured and counted using ImageJ software (NIH, USA). Cell viability (live cells/total cell number) was determined by taking the average cell numbers from three images per sample. Rat and pig ASCs cultured on regular tissue culture plate (TCP) were included as controls for each day. Three samples were measured for each experimental group.

Rat and pig ASCs infiltration through dPMS was observed via Z- stack images captured using confocal microscopy on days 1, 3, and 5. Cells were stained with calcein AM and imaged from top to bottom of the recellularized slices (7 µm apart). The images were stacked using FV1000 software to show all of the cells through the imaged slices. The distance infiltrated by cells was calculated from the number of Z-stack images obtained and the distance between the Z-stacks. All experiments were performed in triplicate.

### Cell proliferation on the dPMS

To check the proliferation of rat and pig ASCs, the number of cells present was assessed on days 1, 3, and 5 for dPMS (n = 3). Total genomic DNA of the cells was isolated using Qiagen DNA mini kit (Qiagen, USA), followed by total DNA quantification with Quant-iT^TM^ PicoGreen dsDNA assay kit (Molecular Probes, USA) according to the manufacturer’s recommendations. Prior to DNA isolation, dPMSs were thoroughly rinsed three times for 15 minutes each with 1X PBS to eliminate any dead cells. The total number of cells for each experimental group was calculated by dividing total DNA concentration with amount of DNA per cell. An equal number of cells cultured on TCP were included as control.

### Immunofluorescence Staining

For immunofluorescence analysis, recellularized dPMSs (on days 3 and 7) and harvested rat hearts were tested. Samples were paraffin embedded following standard protocol and sectioned into 7 μm slices. Paraffin-embedded sections were deparaffinized using xylene and rehydrated by gradation of ethanol. Following rehydration in 1X PBS, heat mediated antigen retrieval with 1X citrate buffer (pH 6.0) was performed using microwave on high power for 15 minutes. The tissue sections were then permeabilized using 0.1% or 0.2% Triton X/PBS solution as needed and blocked with 5% normal goat serum (Abcam, USA) for 45 minutes. All primary and secondary antibodies were diluted to the desired concentration using a blocking solution. The tissue sections were incubated at 4 °C for 18–22 hours with the diluted primary antibodies: von Willebrand Factor (vwf; 1:400), CD31 (1:50), Flt-1 (1:50), VE Cadherin (1:50), CD163 (1:400), CD68 (1:100) and Alpha Smooth Muscle Actin (αSMA; 1:100). After incubation, the tissue sections were thoroughly rinsed with 1X PBS and incubated with appropriate goat derived secondary antibody conjugated with either Alexa Fluor488 or 647 (1:100) for 2 hours at room temperature. Finally, the tissue sections were stained using VECTASHIELD mounting medium with DAPI for nuclei staining. Fluorescence images were captured with an inverted AxioVision A1 microscope (Carl Zeiss). Following the same protocol, negative controls were prepared without addition of primary antibodies.

### Biaxial Mechanical Characterization

Biaxial mechanical tests were performed on decellularized myocardium slices of 300 µm thickness (n = 6), 600 µm thickness (n = 3), and 900 µm thickness (n = 3). Native cardiac slices of 900 µm thickness (n = 3) were also subjected to biaxial testing as a comparison. A 12 mm × 12 mm square was cut with one edge aligned along fiber-preferred direction and the other edge aligned with cross fiber-preferred direction. Biaxial testing method was reported previously with detailed protocols^54–56^. Briefly, four markers were placed in the center of square sample to capture the tissue deformation. Loops of 000 polyester suture of equal length were attached to the sample via stainless steel hooks. Samples were first preconditioned for 10 cycles, and then loaded up to 5 N/m: 5 N/m equibiaxial tension. 5 N/m tension was found to be the maximum tension that the very thin acellular cardiac ECM slices could withstand without sample tear occurring at sites of the hooks (location of stress concentration). It is worthy to point out that we experienced great challenges in testing acellular slices of 300 µm, including large sample variation and many failed tests due to sample tearing. We hence increased the sample size of 300 µm group to n = 6. The biaxial mechanical testing was implemented with the samples completely immersed in PBS (pH 7.4).

### Implantation of dPMS into an acute rat MI Model

All procedures involving animal use, surgeries, and housing were in accordance with the relevant guidelines, regulations, and approval of the Animal Care and Use Committee (IACUC) at the University of Akron (14-11-14-ZRD). Male Sprague Dawley rats (8 weeks old) weighing 250–300 g were purchased (Envigo) and used in these studies. Rats were anesthetized followed by endotracheal intubation. To induce an MI, the pericardium was opened and the left anterior descending (LAD) coronary artery was permanently ligated using a 6–0 polypropylene suture. After an MI has been induced, about 20 minutes after ligation, a 300 μm dPMS was patched to the surface of infarcted area of left ventricle by suturing 3 points around the border. After dPMS implantation, the chest was closed and the animal was left to recover. In total 18 rats were induced with MI and were randomly assigned into either the treatment groups, only dPMS on MI (n = 6), dPMS with labelled ASCs on MI (n = 6), or the control group (MI only; n = 6). One week after surgery, all animals were euthanized and the hearts were harvested for evaluation.

### Statistical analysis

All results are presented as mean ± standard deviation. Statistical analyses were performed using IBM SPSS 24 statistical software. Statistical comparisons were made by one-way analysis of variance (ANOVA) followed by the Kruskal-Wallis non-*parametric test* for between-group differences and *Dunnett’s* correction of the significance level for multiple comparisons. To compare two groups, T test or the Mann–Whitney test (non-*parametric) wa*s performed depending on normal distribution of the data. p values less than 0.05 were considered statistically significant.

## Electronic supplementary material

Supplementary Information

## Data Availability

Primary data used in figures are available on reasonable request.
