# Supplementary material for: A Thin Layer of Decellularized Porcine Myocardium for Cell Delivery
Source: Sci Rep. 2018 Nov 1;8:16206. doi: 10.1038/s41598-018-33946-2 (PMC6212498; doi:10.1038/s41598-018-33946-2)
Supplement: Supplementary file 1 — Supplementary Information [file 41598_2018_33946_MOESM1_ESM.pdf]

Supplementary Information

A Thin Layer of Decellularized Porcine Myocardium for Cell Delivery

Mickey Shah[1, 2], Pawan KC[2], Katherine M. Copeland[3], Jun Liao[3], Ge Zhang[2]*

[1] Integrated Bioscience Program, The University of Akron, Akron, OH 44325-0302, USA

[2] Department of Biomedical Engineering, The University of Akron, Akron, OH 44325-0302, USA

[3] Department of Bioengineering, University of Texas at Arlington, Arlington, Texas 76019, USA

This document contains the following Supplementary Information:

1. **Supplementary Methods**
2. **Cell Attachment Efficiency on dPMS**
3. **Cardiac Related Markers Expression**
4. **GAGs Content Measurement**
5. **Inflammatory Macrophage Staining**
6. **Quantification of Vessels**
7. **Cell Retention Assessment**

1. Supplementary Methods:

Hematoxylin and Eosin Staining

Harvested rat heart tissues, decellularized cardiac tissues, and native cardiac tissues were fixed with 4% paraformaldehyde (w/v) for 3 days at room temperature and then washed with 1X PBS three times for one hour each. Tissues were then dehydrated with graded series of 70%, 95%, and 100% ethanol, followed by two changes of xylenes and three changes of paraffin wax for one hour each using tissue processor (Leica ASP300S; Leica, USA). The processed tissues were embedded in paraffin wax in embedding module and sectioned into 10μm thick slices using a microtome. The tissue slices were deparaffinized with xylene, rehydrated with gradation of ethanol, and stained using Hematoxylin and Eosin kit (H&E; American MasterTech Scientific, USA) according to the manufacturer's recommendations.

Flow Cytometry Analysis of ASCs

The isolated ASCs were stained with stem cell markers and analyzed using flow cytometry. ASCs from the second passage were used for the analysis. For each cell marker, approximately 100,000 ASCs were incubated with either fluorescein isothiocyanate (FITC), alexa fluor 647 (A647) or phycoerythrin (PE) conjugated antibodies for 30 minutes at room temperature protecting from light. The following antibodies were used: FITC conjugated rat antibody for CD90 and CD31, PE conjugated rat antibody for CD29 and CD34, FITC conjugated pig antibody for CD90, CD31 and CD14 (Abcam, USA), and A647 conjugated pig antibody for CD29 (BD Biosciences, USA). Cells were also stained with fluorescent live/dead dye (Life technologies, USA) to obtain the live cell population and only those cells were included in the analysis. The stained cells were subjected to flow cytometer analysis using a LSR Fortessa flow

cytometer (BD Biosciences, USA). Data was analyzed using FlowJo software (v9.8.3). Unstained cells were used with every run for each antibody as a negative control.

Quantiative Real Time Polymerase Chain Reaction (qRT-PCR) Analysis

qRT-PCR was performed to examine the effects of the dPMS on gene expression of rat and pig ASCs. The dPMS reseeded with ASCs was digested using collagenase type II (4 mg/mL, Sigma-Aldrich, USA) at 37℃ for 15 minutes, followed by centrifugation at 600g for 5 minutes to extract cells. Then total RNA was isolated from the cells using an Absolutely RNA Microprep Kit (Agilent Technologies, USA). The total amount and purity of isolated RNA was measured using a Synergy H1 Hybrid Reader nanodrop (Bio-Tek Instruments, USA) and 1% agarose gel electrophoresis. Approximately 40 ng/µl total RNA was converted into cDNA using Verso cDNA kit (Thermo Scientific, USA) per the manufacturer's instructions. The real-time PCR reactions were performed using the SYBR GreenER qPCR SuperMix (Invitrogen, USA) and Applied Biosystems 7500 real-time PCR detection system. Relative gene expression was analyzed with the comparative Ct method.[1] The results were normalized to β-actin and GAPDH and expressed as a fold change for cells isolated from recellularized dPMS compared with cells cultured on TCP (ΔΔCt). qPCR was performed on 4 independent samples and each sample were tested in 3 pseudo-replicates. The primers used in this study are listed in Supplementary Table S1.

Gene	Organism	Forward 5'-3'	Reverse 5'-3'
GAPDH	Rat	TGACAACTTTGGCATCGTGG	CATTGAGAGCAATGCCAGCC
β-Actin	Rat	CCGCGAGTACAACCTTCTTG	CCCATACCCACCATCACACC
αSMA	Rat	CATCACCAACTGGGACGACA	TCCGTTAGCAAGGTCGGATG
vwf	Rat	TCTGGCTGTGACGACTTCAA	GCCATGTCCCTGAGCAAATG
CD34	Rat	GAGACTCAGGGAAAGGCCAAT	GTTCTGTGTCAGCCACCACAT
CD31	Rat	AGTCGTTGTCATTGGAGTGG	TGTTGGAGTTCAGAAGTGGG
GATA4	Rat	CTGTCATCTCACTATGGGCAC	GGCCTGTGATGTCTGAGTG
sox2	Rat	CACATGGCCCAGCACTAC	CCCTCCCAATTCCCTTGTATC
Flt-1	Rat	TTAGGACCAGGAAACAGCACG	GGGTGATCAGCTCCAGGTTT
VE-Cadherin	Rat	AGACACGGGCTGAATACAAG	GCACCGAAAATGTGTATGTGG
SMHC	Rat	ATTGTCTTCCAAGAGTTCCGG	TTGCTCTGCCCAATCCTG
GAPDH	Pig	CAGCAATGCCTCCTGTACCA	GATGCCGAAGTTGTCATGGA
β-Actin	Pig	CACGCCATCCTGCGTCTGGA	AGCACCGTGTTGGCGTAGAG
αSMA	Pig	TGTGACAATGGTTCTGGGCTCTGT	TTCGTCACCCACGTAGCTGTCTTT
vwf	Pig	GTCCTTGCTCCAGCCGCATATTTC	CCCATCATCGTCAACACACTGG
CD34	Pig	GGAAACCACACCAGATGCTT	AGGTCTGAGGCTGGACAGAA
CD31	Pig	CATTTCCAAAGTCAGCAGCA	CATCATCATGCCTCCCTTCT
GATA4	Pig	TGCGTCCCATCAAGACAGAG	TGGCCAGACATGGCACTAAC
sox2	Pig	CAGCTCGCAGACCTACATGA	CTCGGACTTGACCACTGAGC
Flt-1	Pig	TTGGACTGTTGGCACAAAGAC	GCTGTTGCTCGTCAGAATGG
VE-Cadherin	Pig	AAGAACATCGCCCGTGTCAT	CACTGAGCCGATCCAAGGTT
SMHC	Pig	TGGAGGCCGCAGAGTTATTG	ACTGGCTTTGGTTCCGTTGA

Supplementary Table S1. qRT-PCR primer sequences

2. Cell Attachment Efficiency on dPMS: Attachment efficiency of human MSCs, rat ASCs and pig ASCs was assessed on 300 µm dPMS and TCP at 24 hours. Sterilized dPMS was seeded with 200,000 cells on top and 200,000 cells were seeded in 12 well plate as TCP control. After 24 hours, total genomic DNA of the cells was isolated using Qiagen DNA mini kit (Qiagen, USA), followed by total DNA quantification with Quant-iT™ PicoGreen® dsDNA assay kit (Molecular Probes, USA) according to the manufacturer's recommendations. Total number of cells for each experimental group was calculated by dividing total DNA concentration with amount of DNA per cell. The percentage of cell attachment efficiency was calculate using the following equation: % efficiency = (Attached cells / Total seeded cells)*100. We found that 24 hours after cell seeding, the attachment efficiency of rat ASCs, pig ASCs, and human MSCs in TCP were 63.8% ± 1.2%, 66.31% ±6.68, 45.41% ± 12.69 and in dPMSs were 26.5% ± 2.5%, 50.8% ± 16.8%, 33.1% ± 2.4% respectively.

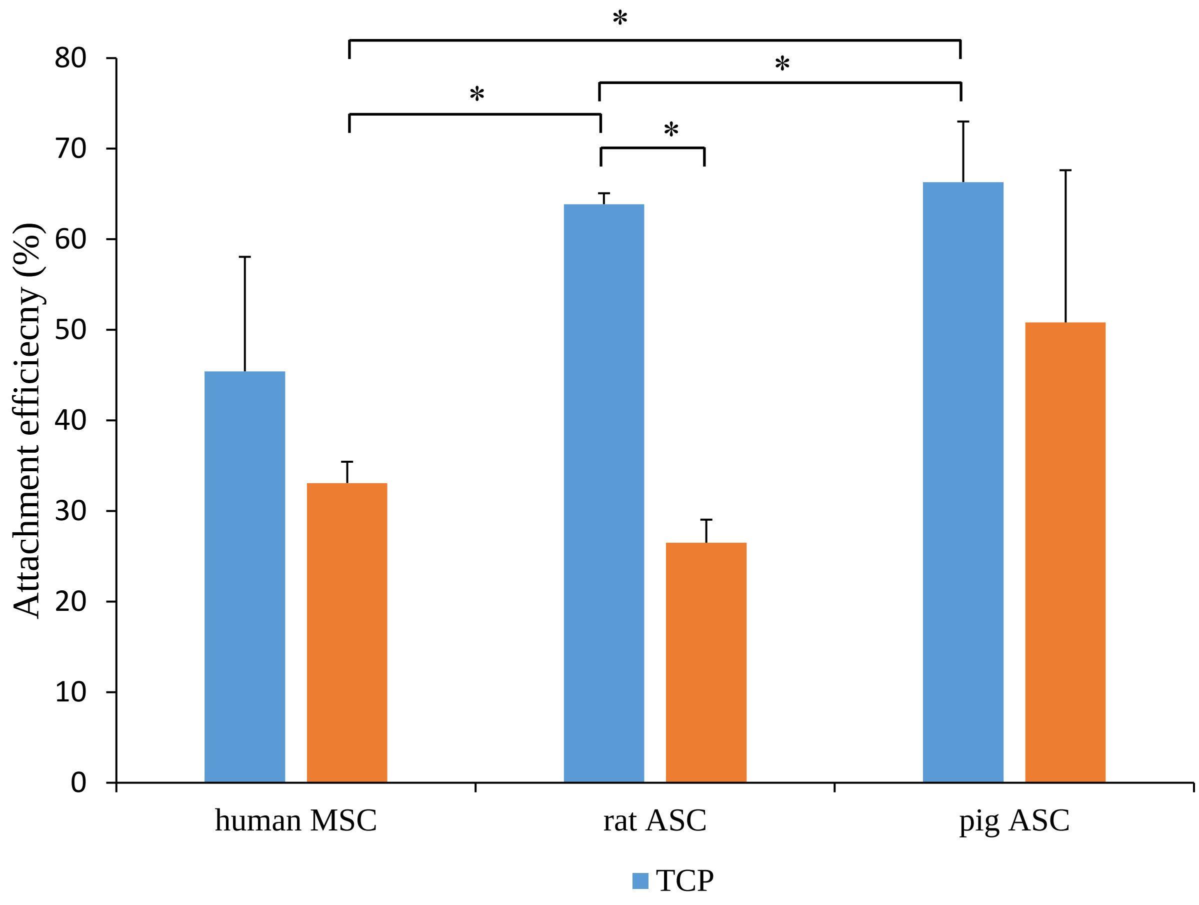

Supplementary Figure S1. Attachment efficiency of human MSCs, rat ASCs and pig ASCs on 300 µm dPMS and TCP at 24 hours. n=3. * represents statistically significant difference between experiment groups with p value <0.05.

3. Cardiac Related Markers Expression: Immunofluorescence staining was performed to detect cardiac related markers expression in rat ASCs cultured on dPMS on day 1, 3, and 5. Briefly, the dPMSs were fixed with 4% paraformaldehyde (w/v) for 2 days and then washed with 1X PBS three times for one hour each. Tissues were then dehydrated with graded series of alcohol, xylene and paraffin wax. The processed tissues were embedded in paraffin wax in embedding module and sectioned into 7-10μm thick slices using a microtome. Paraffin-embedded sections were deparaffinized and heat mediated antigen retrieval with 1X citrate buffer (pH 6.0) was performed using microwave on high power for 15 minutes. The tissue sections were then permeabilized using 0.1% or 0.2% Triton X/PBS solution as needed and blocked with 5% normal goat serum for 45 minutes. All primary and secondary antibodies were diluted to the desired concentration using a blocking solution containing 5% normal goat serum. The tissue sections were incubated at 4°C for 18-22 hours with the diluted primary antibodies: GATA4 conjugated with Alexa Fluor 647 (1:50), cardiac troponin I (1:400), and alpha smooth muscle actin (α-SMA; 1:100). After incubation, the tissue sections were thoroughly rinsed and incubated with secondary antibody conjugated with either Alexa Fluor®488 or 647 (1:100) for 2 hours. Finally, the tissue sections were stained using VECTASHIELD mounting medium with DAPI for nuclei staining. Fluorescence images were captured with an inverted AxioVision A1 microscope (Carl Zeiss). Our results showed that the rat ASCs did not express GATA4 and cardiac troponin I markers on day 1, day 3, or day 5.

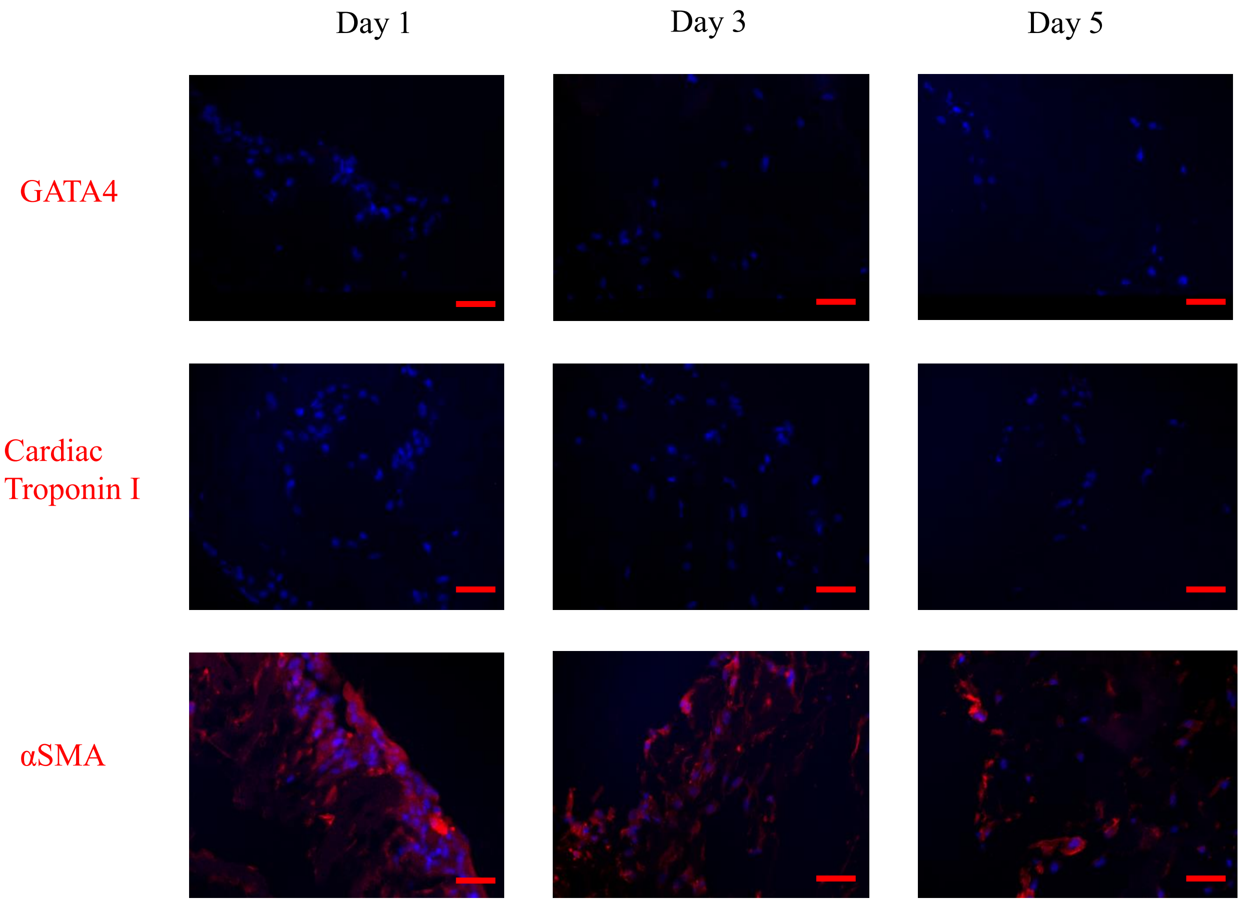

Supplementary Figure S2. Expression of cardiac related markers (GATA4, cardiac troponin and SMA) at day 1, day 3 and day 5 for rat ASCs on 300 µm dPMS. Scale bar 50 µm.

4. GAGs Content Measurement: GAGs content in the native myocardium and decellularized porcine myocardium was quantified using the Blyscan sulfated glycosaminoglycan assay following the manufacturer's instruction (Biocolor, Carrickfergus, UK). The total GAGs content was measured in µg/mg wet tissue using four independent samples. We found that decellularized porcine myocardium had significantly reduced GAGs content (0.22 ± 0.03 µg/mg wet tissue) as compared to native myocardium (2.04 ± 0.51 µg/mg wet tissue).

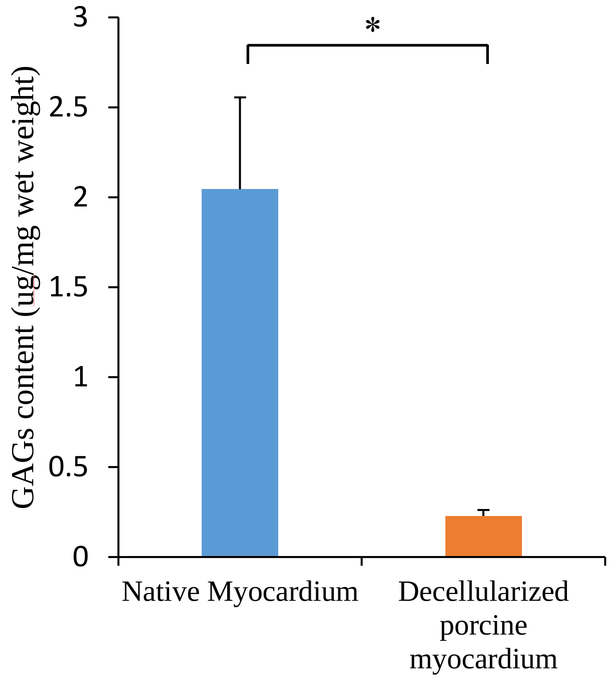

Supplementary Figure S3: GAGs content in native myocardium and decellularized porcine myocardium. n=4. * represents statistically significant difference between experiment groups with p value <0.05.

5. Inflammatory Macrophage Staining: Presence of inflammatory macrophages were checked by immunofluorescence after 1-week in infiltrated host cells in the implanted dPMS. Total macrophages and M2 macrophages were stained by anti–CD 68 and anti-CD 163 antibodies, respectively. We found that some of the infiltrated cells were macrophages (Fig. S4 A). Among them, M2 macrophages also were present (Fig. S4 B). M2 macrophages have been reported to promote positive tissue remodeling.

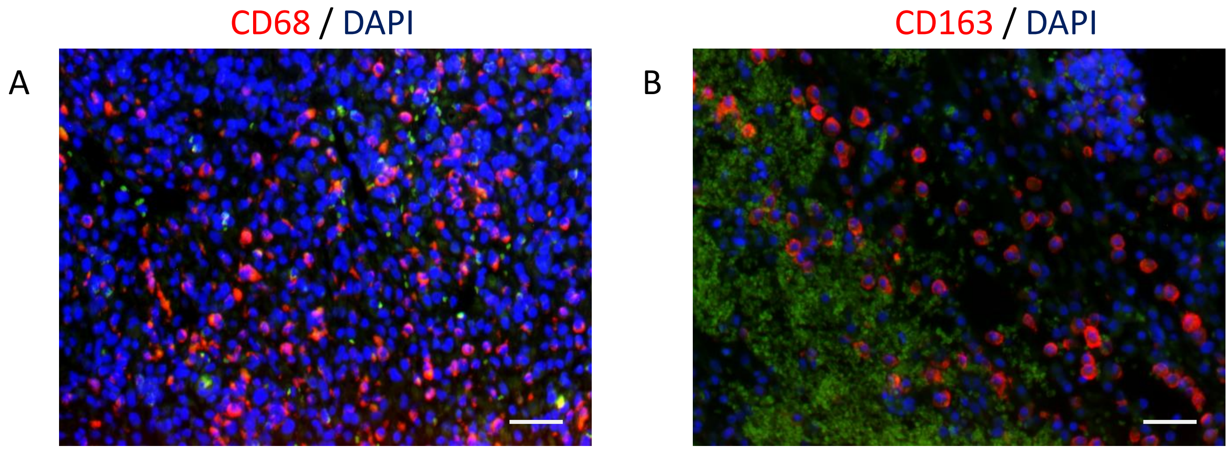

Supplementary Figure S4: Macrophage staining after 1- week of dPMS implantation. Staining of total macrophages (A) and M2 macrophages (B) at infiltrated host cells in grafted dPMS. Scale bar 50 µm.

6. Quantification of the Vessels: The quantification of vessels were carried out in MI group (n=3) and dPMS with ASCs on MI group (n=3), one week post-op. For each slide six regions (four images in infarct area and two images in border region / suture area) were captured at 20X and measured using imagescope software (Leica). Tubular structure positive for αSMA staining were identified as vessel, and further classified into a) arterioles and post capillary veins, b) small artery and venule, c) small veins, d) artery, and e) vein based on their size and shape (Supplementary Table 2) [2]. Average vessels were counted for each region and represented in per mm^2 area. We found the higher number of vessels per mm^2 area in the MI group treated by dPMS with ASCs than the control group (MI only) at week 1 (Fig. S5).

	Size [µm]	Rounded Shape
Arterioles and post capillary veins	5≤ D≤ 10	NA
Small artery and venule	10≤ D≤ 20	NA
Small veins	20≤ D≤ 40	NA
Artery	D ≥ 50	YES
Vein	D ≥ 75	NA

Supplementary Table S2: Classification criteria of the vessels. D: diameter.

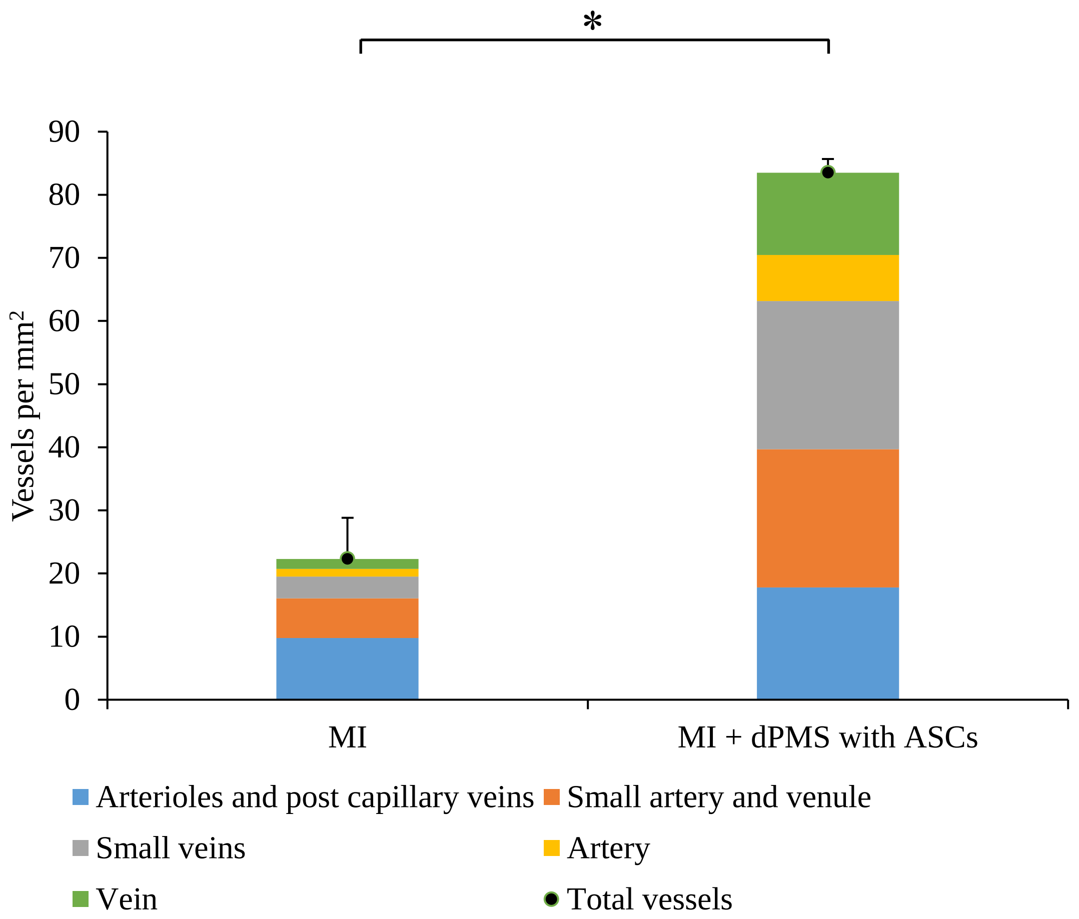

Supplementary Figure S5: Quantification of the vessels in MI group and MI treated by dPMS with ASCs at 1 week post-surgery. n=3. * represents statistically significant difference between experiment groups with p value <0.05.

7. Cell Retention Assessment: The retention of transplanted ASCs using dPMS were examined at 1 week post-op and compared with ASCs delivered by direct injection. CM-Dil labelled ASCs were transplanted to the infarcted area of the rat myocardium either using dPMS or by injection at four spots. 1 week after transplantation, CM-diI stained DAPI+ cells were counted in three images for each rat and normalized to $1mm^2$ area. We found the labelled ASCs in both groups (Fig.S6 A, B) However, a higher number of transplanted cells were identified in the group of MI treated by ASCs delivered using dPMS (Fig. S6C).

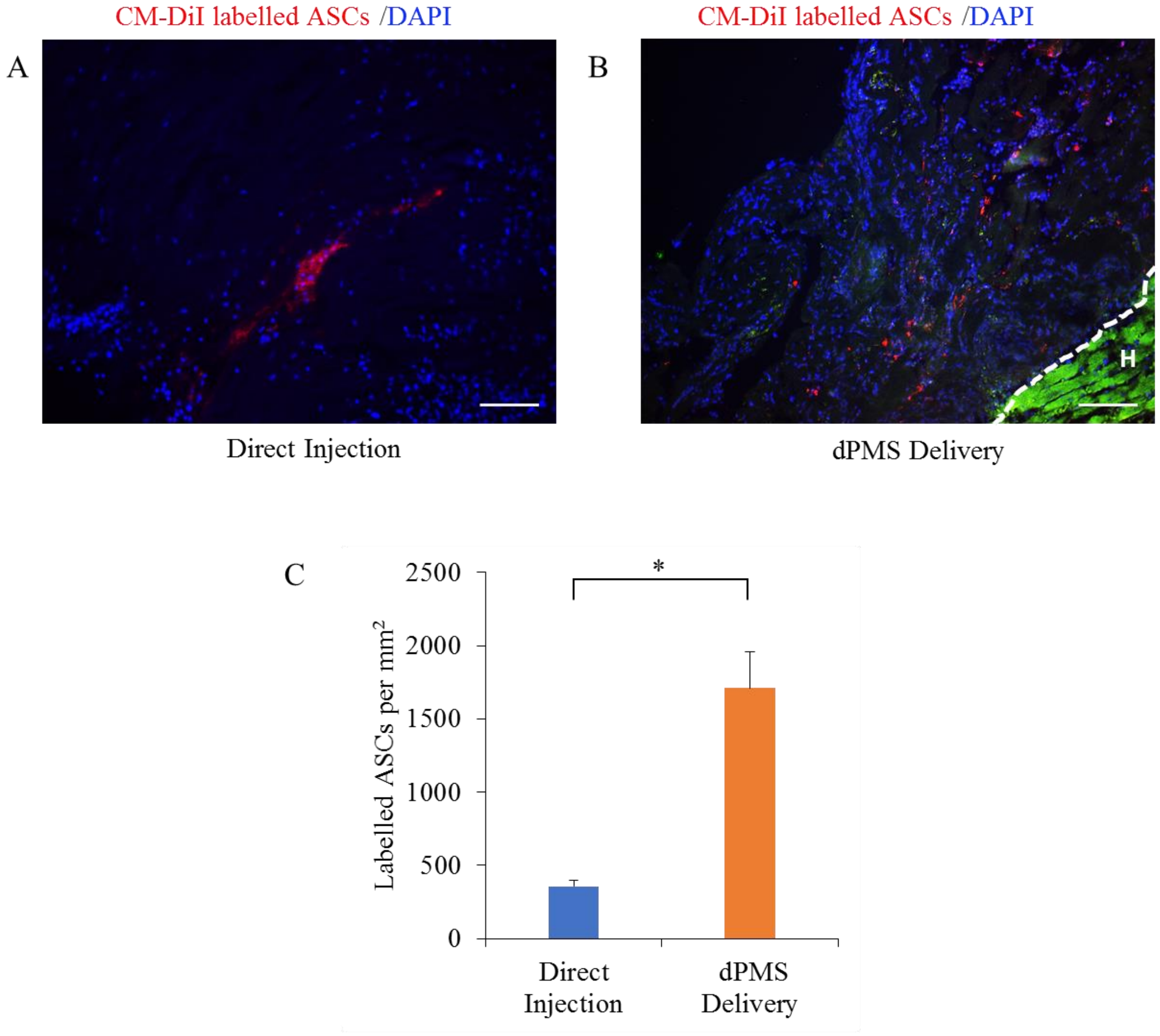

Supplementary Figure S6: The retention of transplanted ASCs 1 week after surgery. Labelled ASCs were found present in the infarcted area of the rat myocardium 1 week after transplantation either by direct injection (A) or using dPMS (B). Dotted line separates dPMS from myocardium. H: Host myocardium. Number of labelled cells present in both the groups were counted in per mm^2 area and compared (C). * represents statistically significant difference between experiment groups with p value <0.05. Scale bar: 100 μm

Reference:

1. Livak, K. J. & Schmittgen, T. D. Analysis of relative gene expression data using real-time quantitative PCR and the 2(-Delta Delta C(T)) Method. *Methods San Diego Calif* **25,** 402–408 (2001).
2. Wiedeman, M. P. Dimensions of blood vessels from distributing artery to collecting vein. *Circ. Res.* **12,** 375–378 (1963).
